# Bioinformatics delimitation of the psychrophilic and psychrotolerant actinobacteria isolated from the Polar Frontal waters of the Southern Ocean

**DOI:** 10.1016/j.dib.2018.03.014

**Published:** 2018-03-08

**Authors:** Palaniappan Sivasankar, Bhagwan Rekadwad, Subramaniam Poongodi, Kannan Sivakumar, Bhaskar Venkateswaran Parli, N. Anil Kumar

**Affiliations:** a Department of Environmental Science, Periyar University, Periyar Palkalai Nagar, Salem 636011, Tamil Nadu, India; b National Centre for Microbial Resource, National Centre for Cell Science, Pune 411021, India; c Centre of Advanced Study in Marine Biology, Faculty of Marine Sciences, Annamalai University, Parangipettai 608502, Tamil Nadu, India; d National Centre for Antarctic and Ocean Research, Headland Sada, Vasco-da-Gama, Goa 403804, India

**Keywords:** Psychrophile, Psychrotolerant, Antarctic Ocean, Polar Front, Marine Actinobacteria

## Abstract

Identification of microorganisms plays a key role in the determination of the composition of microbial diversity for bioprospecting of biotechnologically important biomolecules. Digitalization is the process that solve discrepancies in microbial identification and cataloguing their diversity in distinct ecological habitats. In view of this connection, the psychrophilic and psychrotolerant actinobacteria were isolated from the water samples of the Polar Frontal region of the Southern Ocean. 16S rRNA gene sequencing for identification of psychrophiles was carried out and sequences were deposited in NCBI GeneBank. 16S rRNA gene sequences were used to create QR codes, CGR, FCGR and GC plot. This generated digital data help to relate the diversity amongst the isolated actinobacterial strains. The digital data showed considerable divergence among the actinobacterial strains. This generated bioinformatics data is helpful in the delimitation of the psychrophilic and psychrotolerant actinobacteria. Thus, the present study is a robust and accurate method for the identification of Polar microorganisms in a fixed boundary. Hence, this work will help to assign a unique digital identity to microorganisms in near future [9-19].

**Specification table**

**Table:** 

*Subject area*	Marine Microbiology
*More specific subject area*	Polar Microbiology
*Type of data*	Quick Response (QR) codes, Chaos Game Representation (CGR), Chaos Game Representation Frequencies (FCGR) and GC percentage graph
*How data was acquired*	Wet lab isolation, 16S rRNA gene sequencing, and bioinformatics analysis
*Data format*	Raw and analysed
*Experimental factors*	Bioinformatics tools were used for analysis
*Experimental features*	Polar Frontal region (PF1–53°07′90″S; 47°48′061″E and PF2–56°29′956″S; 54°41′213″E), Southern Ocean
*Data source location*	Repository of Marine Actinobacteria, Centre of Advanced Study in Marine Biology, Faculty of Marine Sciences, Annamalai University, Parangipettai-608 502, Tamil Nadu, India
*Data accessibility*	Data available within this paper

**Value of the data**

• Bioinformatics data of the psychrophilic and psychrotolerant actinobacteria of the Polar Frontal waters have significant importance in the biodiversity and biotechnology of microorganisms found in the polar regions and other cold environments.
• An earnest attempt was made to digitize the 16S rRNA gene sequence of the psychrophilic and psychrotolerant actinobacteria of the Polar Frontal waters of the Antarctic Ocean.
• The work is also significant on the score that it helps to build a database on microbial communities of Antarctica and to assign a unique digital identity to microorganisms.

## 1 Experimental design, materials and methods

Seawater samples were collected during the 7th Indian Scientific Expedition to the Indian Ocean Sector of the Southern Ocean (SOE-2012-13). The samples were collected at two sampling stations *viz*., Polar Front-1 (53°07′90″S; 47°48′061″E) and Polar Front-2 (56°29′956″S; 54°41′213″E) using CTD (SEABIRD 911 plus, USA). The isolation of psychrophilic and psychrotolerant actinobacteria was done following the recommended protocol [1].

The actinobacterial strains were identified based on their morphological (aerial mass colour, melanoid, reverse side and soluble pigments), physiological (carbon source assimilation), and chemo-taxonomical characteristics (cell wall amino acid and whole-cell sugar) by following the recommended method of Shirling and Gottlieb [2], and Lechevalier and Lechevalier [3]. The actinobacterial strains were warranted at genus level by comparing the data with the identification key developed by Nonomura [4].

16S rRNA gene sequencing was performed to identify the taxonomic position of the actinobacterial strains. Genomic DNA was extracted [5] and amplified using using the universal bacterial primers 27f (5′-GAGTTTGATCCTGGCTCAG-3′) and 1492r (5′-TACGGCTACCTTGTTACGACTT-3′) following the PCR conditions described by Karuppiah et al. [6]. The amplified products were purified using QIA quick PCR cleanup kit (Qiagen Inc., Chatsworth) and were sequenced using ABI automated sequencer (Applied Biosystems-3100) at Macrogen Inc., Republic of Korea. The forward and reverse sequences were assembled using EZ-Taxon database (https://www.ezbiocloud.net) and the sequence similarity was tested in the BLASTn program of the NCBI-GenBank database (http://www.ncbi.nlm.nih.gov/BLAST/). The phylogenetic tree was constructed to understand the actinobacterial lineage using the neighbour-joining method of Saitou and Nei [7]. The topology of the phylogenetic tree was evaluated, using the bootstrap resampling method of Felsenstein [8] with 1000 replicates.

## 2 Data

Nine 16S rRNA gene sequences of psychrophilic (PSY13, PSY15, PSY21, and PSY25) and psychrotolerant (PST1, PST2, PST3, PST4, and PST5) actinobacteria were submitted to NCBI Gen Bank database under the accession numbers KY120275-KY120283. The digitization of the actinobacterial sequences was carried as per algorithm and guidelines developed by Rekadwad et al., [9], [10], [11], [12], [13], [14], [15], [16], [17], [18], [19].

**Fig. 1:**
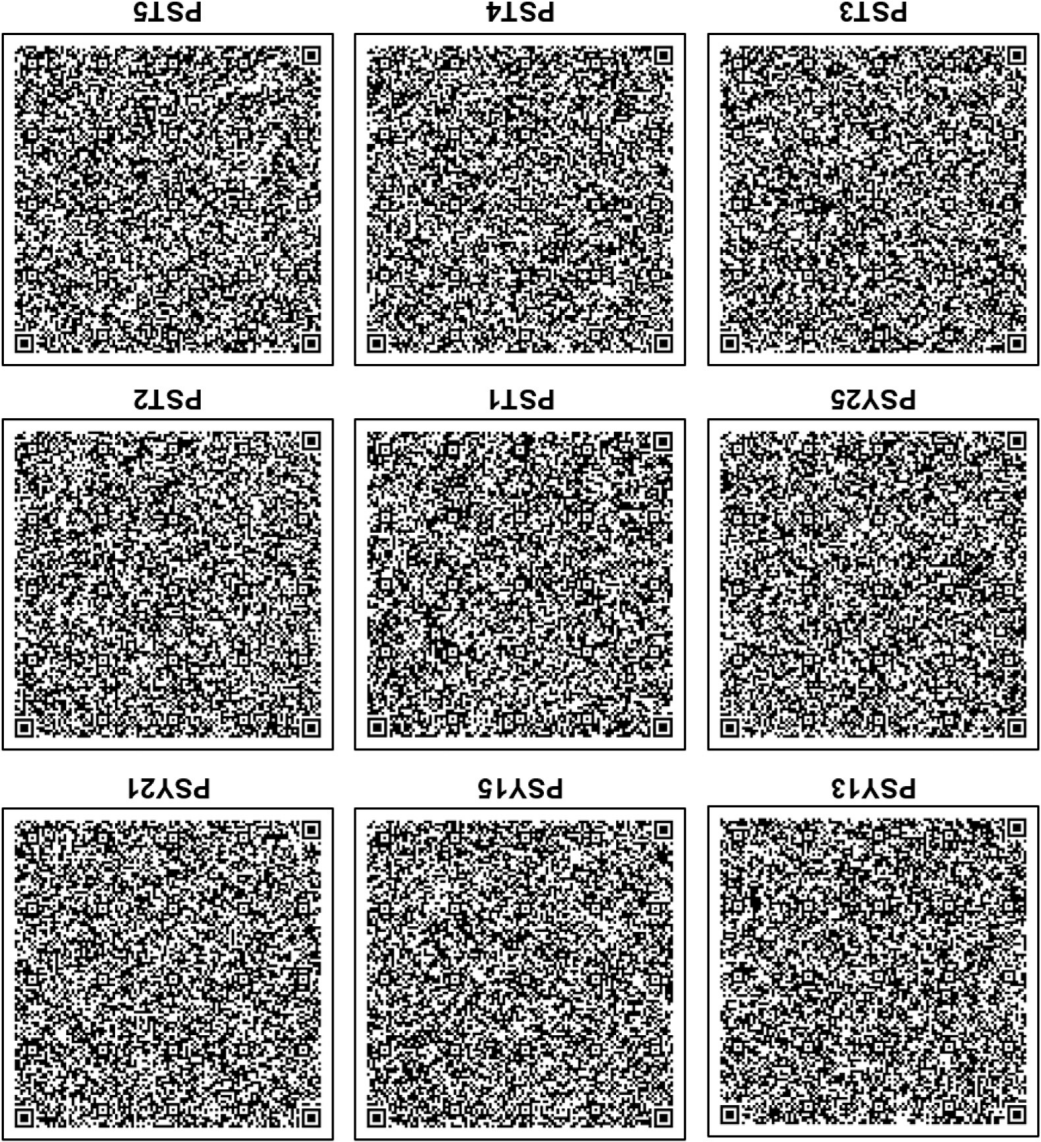
Quick Response (QR) codes of 16S rRNA gene sequences of the psychrophilic and psychrotolerant actinobacteria.

**Fig. 2:**
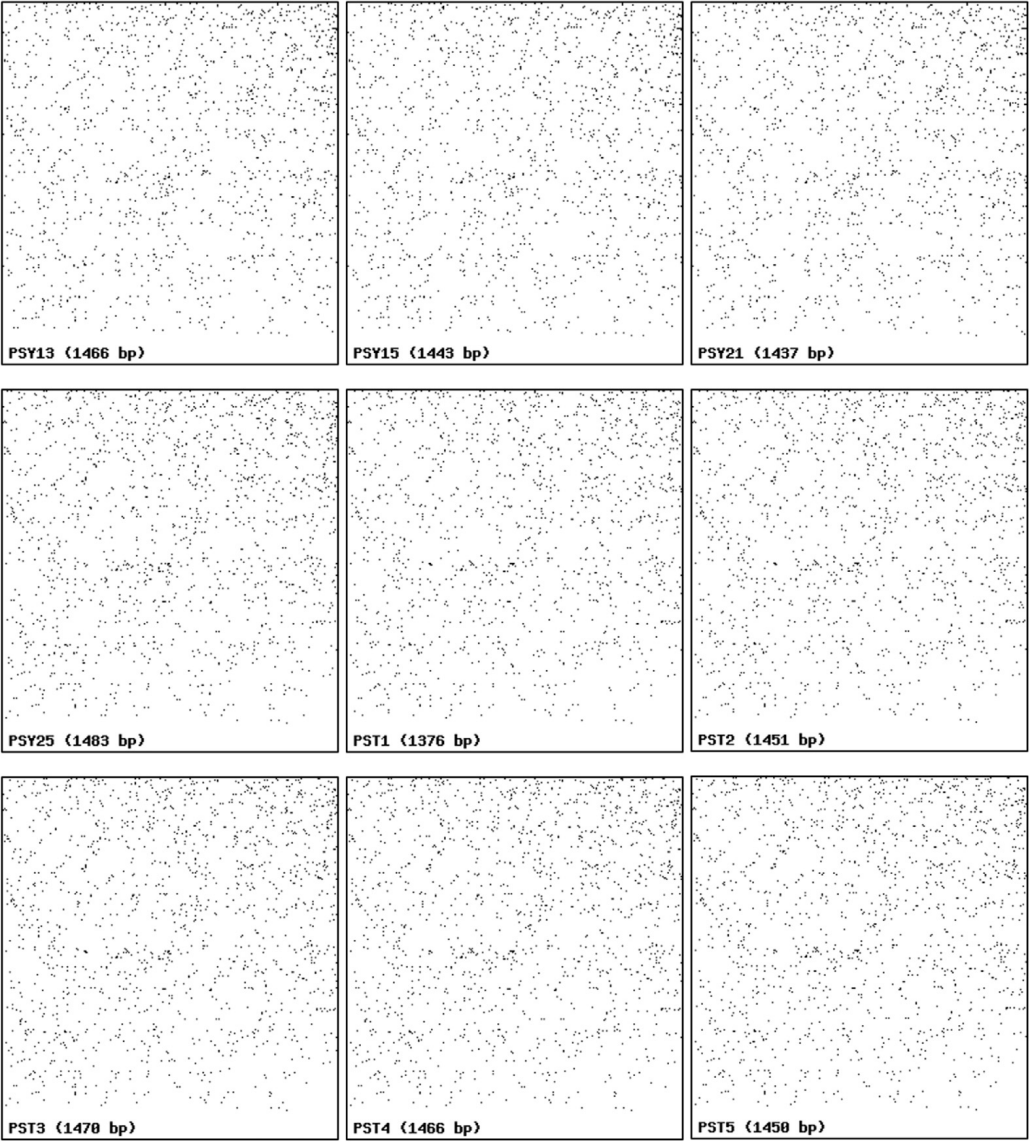
Chaos Game Representation (CGR) of 16S rRNA gene sequences of the psychrophilic and psychrotolerant actinobacteria.

**Fig. 3:**
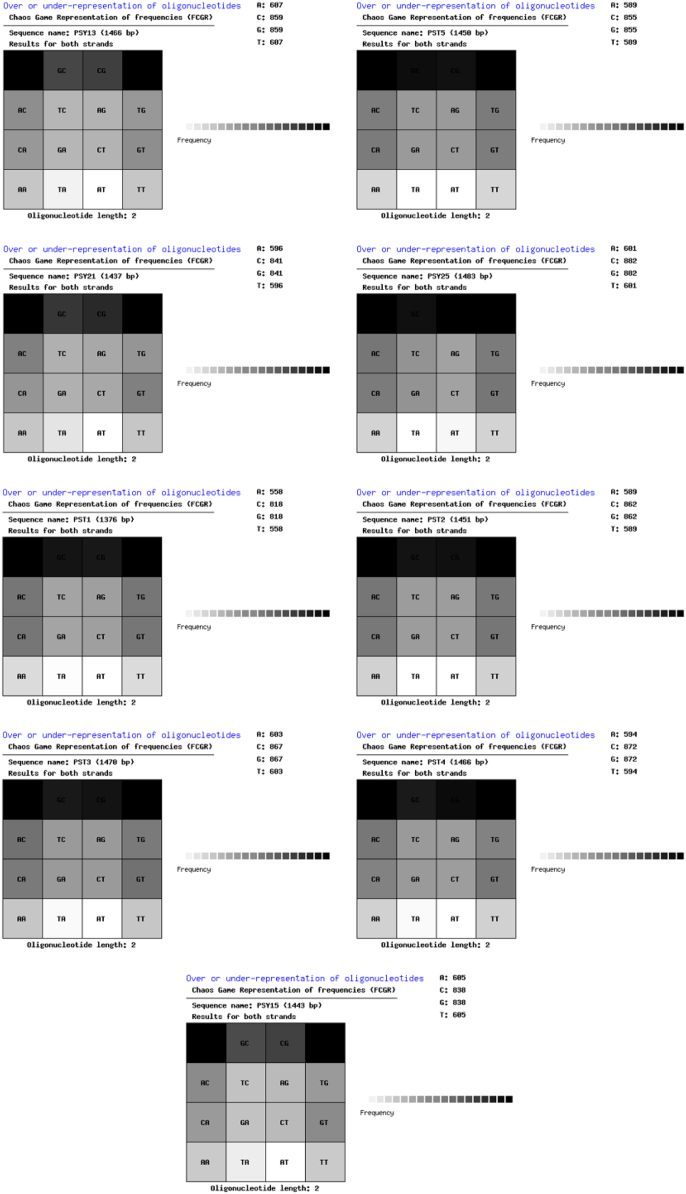
Chaos Game Representation of Frequencies (FCGR) of 16S rRNA gene sequences of the psychrophilic and psychrotolerant actinobacteria.

**Fig. 4:**
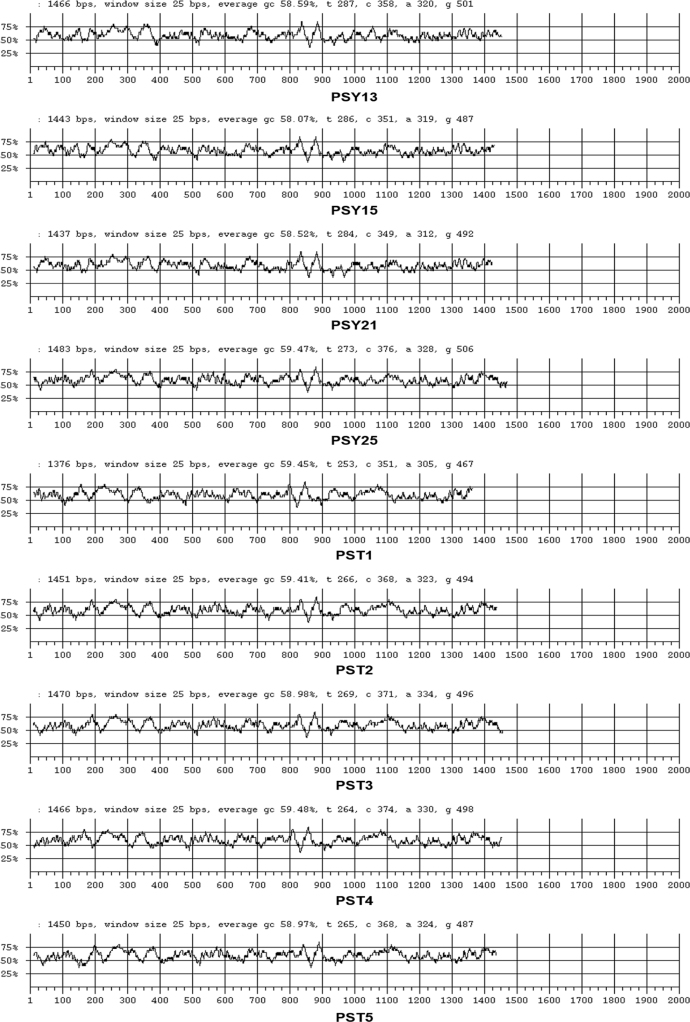
Graphical representations of G + C content of 16S rRNA gene sequences of the psychrophilic and psychrotolerant actinobacteria.

QR codes were generated through QR Code Generator^Pro^ tool (Fig. 1) and the unique barcode sequences were retried using DNA BarID (http://www.neeri.res.in/DNA_BarID/DNA_BarID.htm) (Table 1). The Chaos Game Representation (CGR) and Chaos Game Representation Frequencies (FCGR) were digitally presented using the open source BioPHP bioinformatics tool (Fig. 2, Fig. 3). The graphical representation of the G+C content of the 16S rRNA gene sequences of the psychrophilic and psychrotolerant (Fig. 4 and Table 2) was done using the Webgenetics tool (https://www.webgenetics.com/acts/wg?prog=gcplot).

**Table 1:** Digital data harbouring the unique barcode sequences of the psychrophilic and psychrotolerant actinobacteria.

**Accession no.**	**Sequence length**	**Barcode start**	**Barcode end**	**Unique barcode sequences**
KY120275	1466	71	140	CACGTGAGCAACCTGCCCCTGACTCTGGGAAATTCCTAAGCGGTGGAAACGCCGTCTAATACCGGATACG
KY120276	1443	71	140	ACACGTGAGCAACCTGCCCCTGACTCCGGGATAAGCGGTGGAAACGCCGTCTAATACCGGATACGCCGCC
KY120277	1437	71	140	TAACACGTGAGCAACCTGCCCCTGACTCTGGGATAAGCGGTGGAAACGCCGTCTAATACCGGATACGACC
KY120278	1483	71	140	GCGAACGGGTGAACACGTGGGCAATCTGCCCTGCACTCTGGGACAAGCCCTGGAAACGGGCTAATACCGG
KY120279	1376	71	140	CTCTGGGACAAGCCCTGGAAACGGGGTCTAATACCGGATATGACTGTCCATCGCATGGTGGATGGTGTAA
KY120280	1451	71	140	ACGGGTGAGTAACACGTGGGCAACCTGCCCTGCACTCTGGGACAAGCCCTGGAAACGGGGTCTGGCACTA
KY120281	1470	71	140	CCGGGTGAGTAACACGTGGGCAATCTGCCCAGCACTCTGGGTCAAGCCCTGGAAACGGGGTCTAAGACAA
KY120282	1466	71	140	CTGCCCTGCACTCTGGGACAAGCCCTGGAAACGGGGTCTAATACCGGATACTGACCCGCTTGGGCATCCA
KY120283	1450	71	140	TAGTGGCGAACGGGTGAGTAACACGTGGGCAATCTGCCCTGCACTCTGGGACAAGCCCTGGAAACGGGGT

**Table 2:** G+C percentage of 16S rRNA gene sequences of the psychrophilic and psychrotolerant actinobacteria.

**Accession number.**	**Species**	**Strain designation**	**Average G+C content (%)**
KY120275	*Nocardiopsis dassonvillei*	PSY13	58.59
KY120276	*Nocardiopsis prasina*	PSY15	58.07
KY120277	*Nocardiopsis alba*	PSY21	58.52
KY120278	*Streptomyces albus*	PSY25	59.47
KY120279	*Streptomyces albidoflavus*	PST1	59.45
KY120280	*Streptomyces exfoliatus*	PST2	59.41
KY120281	*Streptomyces pactum*	PST3	58.98
KY120282	*Streptomyces griseorubens*	PST4	59.48
KY120283	*Streptomyces althioticus*	PST5	58.97

## Appendix A Supplementary material

Fig. S4. Consensus trees of 18 selected NUMTs.

## References

[1] Lo Giudice A., Brilli M., Bruni V., De Domenico M., Fani R. (2007). Michaud bacterium-bacterium inhibitory interactions among psychrotrophic bacteria isolated from Antarctic seawater (Terra Nova Bay, Ross Sea). FEMS Microbiol. Ecol..

[2] Shirling E.B., Gottlieb D. (1966). Methods for characterization of Streptomycetes species. Int. J. Syst. Bacteriol..

[3] Lechevalier M.P., Lechevalier H. (1970). Chemical composition as a criterion in the classification of aerobic actinomycetes. Int. J. Syst. Bacteriol..

[4] Nonomura H. (1974). Key for classification and identification of 458 species of the Streptomycetes included in ISP. J. Ferment. Technol..

[5] Everest G.J., le Roes-Hill M., Omorogie C., Cheung S.K., Cook A.E., Goodwin C.M., Meyers P.R. (2013). *Amycolatopsis umgeniensis* sp. nov., isolated from soil from the banks of the Umgeni River in South Africa. Antonie Van Leeuwenhoek.

[6] Karuppiah V., Aarthi C., Sivakumar K. (2011). Enhancement of PCR amplification of actinobacterial 16S rRNA gene using an adjuvant, dimethyl sulfoxide. Curr. Sci..

[7] Saitou N., Nei M. (1987). The neighbour-joining method: a new method for reconstructing phylogenetic trees. Mol. Biol. Evol..

[8] Felsenstein J. (1985). Confidence limits on phylogenies: an approach using the bootstrap. Evolution.

[9] Rekadwad BN B.N., Khobragade C.N. (2017). Data on graphical representation (CGR and FCGR) of bacterial and archaeal species from two Soda Lakes. Data Brief.

[10] B.N. Rekadwad BN, J.M. Gonzalez, New Generation DNA Sequencing (NGS): Mining for genes and the potential of extremophiles, in: V.C. Kalia, P. Kumar (eds.), Microbial Applications Vol.1 - Bioremediation and Bioenergy, Springer International Publishing Switzerland, AG, Springer Nature, 2017, pp. 255–268. 〈 10.1007/978-3-319-52666-9_12〉.

[11] B.N. Rekadwad BN, J.M. Gonzalez, C.N. Khobragade, Functional diversity and applications of mobile group II introns, in: G. Arora, A. Sajid, V.C. Kalia (eds.), Drug Resistance in Bacteria, Fungi, Malaria, and Cancer, Springer International Publishing Switzerland, AG, Springer Nature, 2017, pp. 161–169. 〈 10.1007/978-3-319-48683-3_6〉.

[12] Rekadwad B.N., Khobragade C.N. (2016). Digital data for quick response (QR) codes of alkalophilic *Bacillus pumilus* to identify and to compare bacilli isolated from Lonar Crator Lake, India. Data Brief.

[13] Rekadwad B.N., Gonzalez J.M. (2017). Correcting names of bacteria deposited in National Microbial Repositories: an analysed sequence data necessary for taxonomic re-categorization of misidentified and misclassified bacteria-ONE example, genus *Lysinibacillus*. Data Brief.

[14] Rekadwad B.N., Khobragade C.N. (2016). Digital data for Quick Response (QR) codes of thermophiles to identify and compare the bacterial species isolated from Unkeshwar hot springs (India). Data Brief.

[15] Rekadwad B.N., Gonzalez J.M., Khobragade C.N. (2016). Genomic analysis of marine bacterium: a bioinformatics for comparison, evaluation and interpretation of DNA sequences. Biomed. Res. Int..

[16] Rekadwad B.N., Khobragade C.N. (2016). Digital data of quality control strains under general deposit at Microbial Culture Collection (MCC), NCCS, Pune, India: a bioinformatics approach. Data Brief.

[17] Rekadwad B.N., Khobragade C.N. (2016). Determination of GC content of *Thermotoga**maritima*, *Thermotoga**neapolitana* and *Thermotoga**thermarum* strains: a GC dataset for higher level hierarchical classification. Data Brief.

[18] Rekadwad B.N., Khobragade C.N. (2016). Data on true tRNA diversity among uncultured and bacterial strains. Data Brief.

[19] Rekadwad B.N., Khobragade C.N. (2016). Bioinformatics data supporting revelatory diversity of cultivable thermophiles isolated and identified from two terrestrial hot springs, Unkeshwar, India. Data Brief.

